# Distributed acoustic sensing for active offshore shear wave profiling

**DOI:** 10.1038/s41598-022-13962-z

**Published:** 2022-06-11

**Authors:** Andrew Trafford, Robert Ellwood, Loris Wacquier, Alastair Godfrey, Chris Minto, Mark Coughlan, Shane Donohue

**Affiliations:** 1 School of Civil Engineering, University College Dublin, Dublin, Ireland; 2 SFI Research Centre in Applied Geosciences (iCRAG), Dublin, Ireland; 3 Optasense Limited, Farnborough, Hampshire, UK

**Keywords:** Civil engineering, Energy infrastructure, Energy infrastructure, Geophysics

## Abstract

The long-term sustainability of the offshore wind industry requires the development of appropriate investigative methods to enable less conservative and more cost-effective geotechnical engineering design. Here we describe the novel use of distributed acoustic sensing (DAS) as part of an integrated approach for the geophysical and geotechnical assessment of the shallow subsurface for offshore construction. DAS was used to acquire active Scholte-wave seismic data at several locations in the vicinity of a planned windfarm development near Dundalk Bay, Irish Sea. Complimentary additional datasets include high-resolution sparker seismic reflection, cone penetration test (CPT) data and gravity coring. In terms of fibre optic cable selection, a CST armoured cable provided a reasonable compromise between performance and reliability in the offshore environment. Also, when used as a seismic source, a gravity corer enabled the fundamental mode Scholte-wave to be better resolved than an airgun, and may be more suitable in environmentally sensitive areas. Overall, the DAS approach was found to be effective at rapidly determining shear wave velocity profiles in areas of differing geological context, with metre scale spatial sampling, over multi-kilometre scale distances. The application of this approach has the potential to considerably reduce design uncertainty and ultimately reduce levelised costs of offshore wind power generation.

## Introduction

Offshore wind is a fundamental part of our plans for a green energy future. Reducing the Levelised Cost of Electricity (LCOE) is important for ensuring the future sustainability of wind energy^1–3^. As the turbine foundation adds significantly to overall capital costs, the application of more cost effective and less conservative geotechnical engineering is essential to the longer-term sustainability of the industry^3,4^. Engineers, however, often have to resort to conservative designs due to a lack of available subsurface information^5^. Current best practice for the characterisation of geotechnical properties of shallow marine sediments typically involves shallow seismic investigations and the subsequent collection of targeted borehole and/ or Cone Penetration Test (CPT) data^6^. This approach requires multiple phases of high-cost survey work, with potentially low acquisition rates of sparse data, requiring technical integration that has limitations for design.

Over recent years, there has been increased recognition from the geotechnical industry on the use of geophysical properties when designing these structures^4,7–9^. It is generally accepted that the dynamic loading applied to establish the natural frequency of the structure induces very small displacements^10^. Therefore, the small-strain stiffness of the soil can be used to model soil displacements and is recognized as a key parameter when designing these dynamically sensitive structures^4,10,11^. The seismic shear wave velocity (V_s_) is related, through elastic theory, to the small strain shear modulus G_max_, which is a critical input parameter for several engineering applications, including static and dynamic analysis of offshore foundation systems, soil liquefaction analysis and input for advanced constitutive soil models. Its measurement has, therefore, been recommended as standard practice for offshore site investigations in order to reduce design uncertainty and ultimately reduce the LCOE of such projects^8^.

In this study, we present an approach for rapid acquisition of spatially extensive V_s_ data based on Distributed Acoustic Sensing (DAS) of fibre optic cables laid on the seabed. Using this approach, standard telecommunication optical fibres are interrogated by transmitting a temporally short light pulse (in the order of 10 ns) and analysing the backscatter from microstructural variations naturally present within the fibre^12^. Depending on the power level of the propagating pulse, this effect is dominated primarily by elastic Rayleigh backscatter. Approximately 0.1% of the backscattered light is captured by the fibre and returned to the interrogator unit for analysis^13^. By understanding the propagation velocity of light within the silica fibre^14^ and applying precise time gating it is possible to interrogate the characteristics of the light returning from different positions along the fibre. A range of techniques have been developed^15,16^ to encode each pulse with additional information that enable instantaneous relative quantitative strain measurements to be made at each location along the sensing cable. Generating multiple successive pulses of light allows the strain at each location to be interrogated several thousand times a second, enabling the detection of seismically induced strain within the fibre^17,18^. In this study, seabed V_s_ profiles are produced following inversion of DAS derived seismic Scholte wave dispersion data. Scholte waves propagate at the water–solid seabed interface and are different to Rayleigh waves propagating at the free surface, with the velocity variations related to the wavelength to water depth ratio^19–21^.

### Geological setting

**Figure 1:**
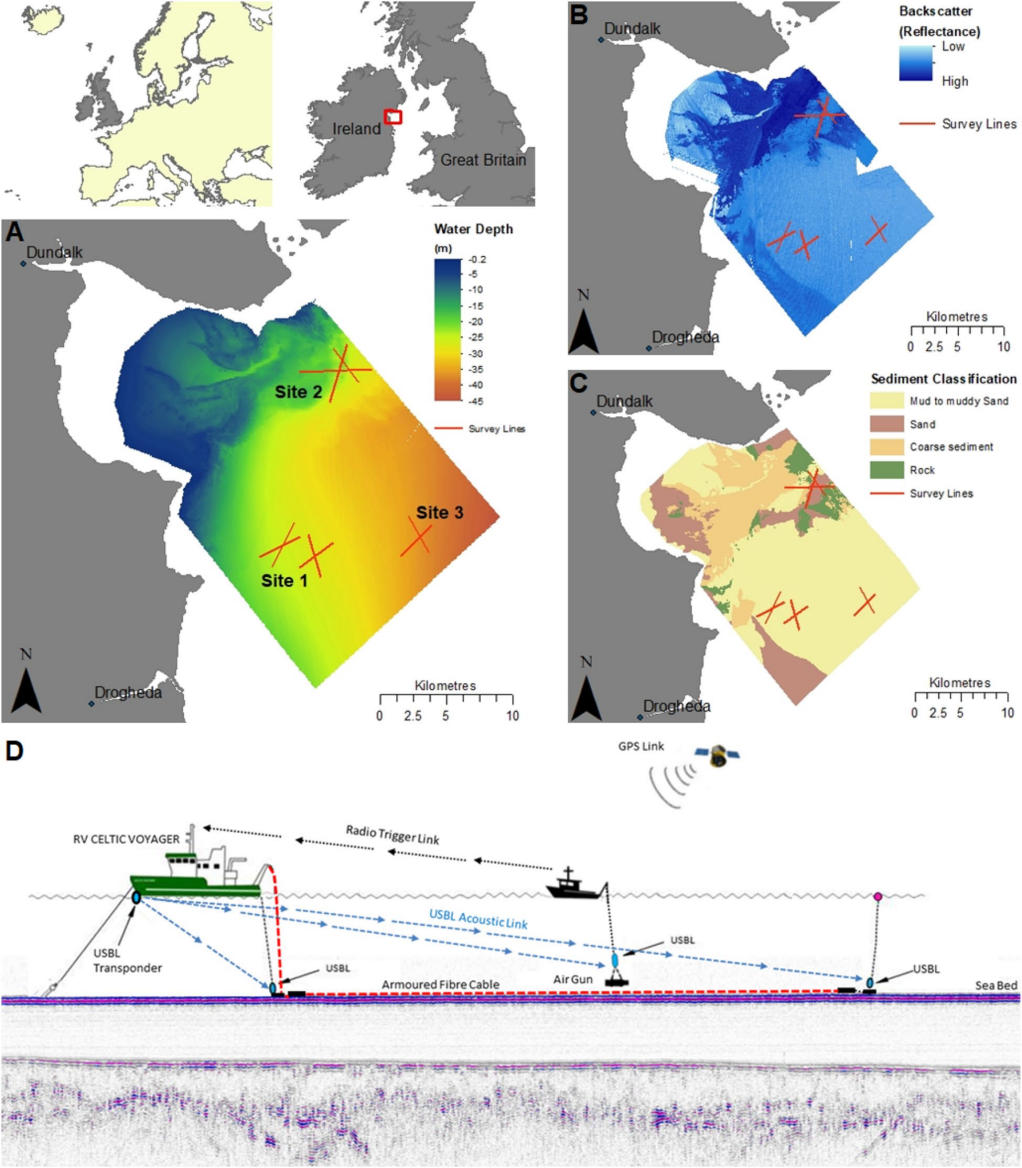
Site map showing (**a**) bathymetry derived from multibeam echosounder (MBES) data, (**b**) acoustic backscatter derived from MBES data, (**c**) sediment classification with test locations, and (d) schematic view of deployment methodology overlaid on high resolution sparker reflection data (Not To Scale). MBES and sediment classification data is Irish Public Sector Data (INFOMAR) licensed under a Creative Commons Attribution 4.0 International (CC BY 4.0) licence and accessed through https://www.infomar.ie. The maps were generated using ArcGIS Desktop v10.7 (www.arcgis.com).

The survey locations were chosen at positions where previous geotechnical data was available in order to confirm and correlate the findings from the DAS survey approach. The locations spanned differing seabed conditions with thick sequences of very soft clay/silts known as the Western Irish Sea Mud Belt^22^ (WISMB; Sites 1 and 3) to the south and areas of shallow glacial deposits, including glacial till, (Site 2) to the north, in water depths between c. 20 and 50 m (Fig. 1). Data are presented here from Sites 1 and 2. The WISMB covers an extensive area with water depths up to 100 m that is filled with substantial thicknesses (up to 40 m) of marine, Holocene sediments^22^. Previous investigations^23–25^ in the area have identified four stratigraphic units in the WISMB consisting of a basal subglacial (lodgement) till (Unit 4) emplaced by the Irish Sea Ice Stream (ISIS) as it advanced across the area. Unit 4 overlies irregular bedrock. As the ISIS retreated during deglaciation, glaciomarine to glaciolacustrine, ice-proximal outwash gravels, sands and silts were deposited (Unit 3). As deglaciation continued, the area became increasingly ice-proximal and dark muddy sands (Unit 2) were deposited in a glaciomarine to the marine environment. Finally, Holocene marine muds (Unit 1) varying in thickness (up to 27 m) lie above an erosive horizon and form the contemporary seabed. The area is economically important being extensively trawled for the Dublin Bay prawns (*Nephrops norvegicus*) and is also earmarked for potential offshore windfarm developments^26^.

## Results

### Active Scholte wave acquisition using DAS

The Distributed Acoustic Sensing was carried out using an OptaSense ODH4 interrogator unit housed within the ‘dry lab’ onboard the Marine Institutes’ research vessel Celtic Voyager. In order to give increased data redundancy, the 1000 m long fibre optic cable was configured with a spliced return at the distal end resulting in 2000 m of active fibre being interrogated. The cable was housed on a mechanical winch on the back deck and deployed using a 9 m Rigid Hull Inflatable Boat (RHIB) which also acted as the source vessel (Fig. 1d). The distal end of the cable was secured to the seabed using a weight and buoy for sighting and retrieval. Both cable ends were mechanically isolated from the retrieval buoys by a double weight system to prevent swell noise introducing unwanted strain on the cable. The DAS system was configured to acquire data using a 2 m gauge length with a channel spacing of 1 m, effectively creating 2000 overlapping active channels. In this configuration the system can acquire up to 5 km of data with no loss in data integrity. For the majority of the survey the source energy was supplied by an 12 cu in Sercel Mini G Airgun. A Geo-Source 400 sparker seismic system was used to provide complimentary sub bottom profiling data. Further details of the methodology is provided in the Methods section, at the end of this paper.

**Figure 2:**
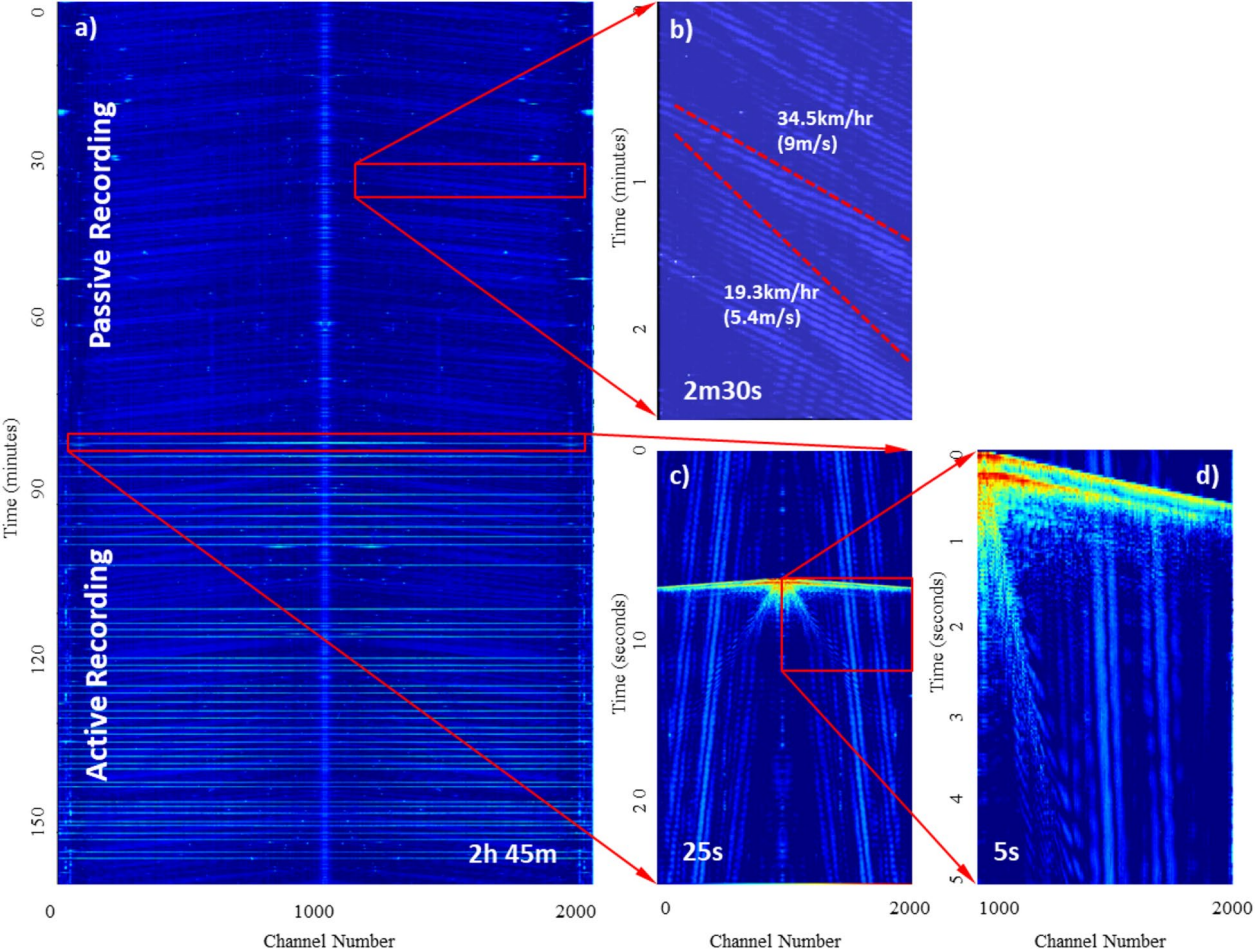
DAS data from site 1 showing (**a**) 2 h 45 min waterfall FBE data with passive and active data recording. (**b**) 2.5 min passive data showing ocean pressure waves, with apparent group wave velocities highlighted (red dashed lines). (**c**) 25 s active data showing airgun shot with pressure wave interference. (**d**) 5 s shot record on 900 channels.

**Figure 3:**
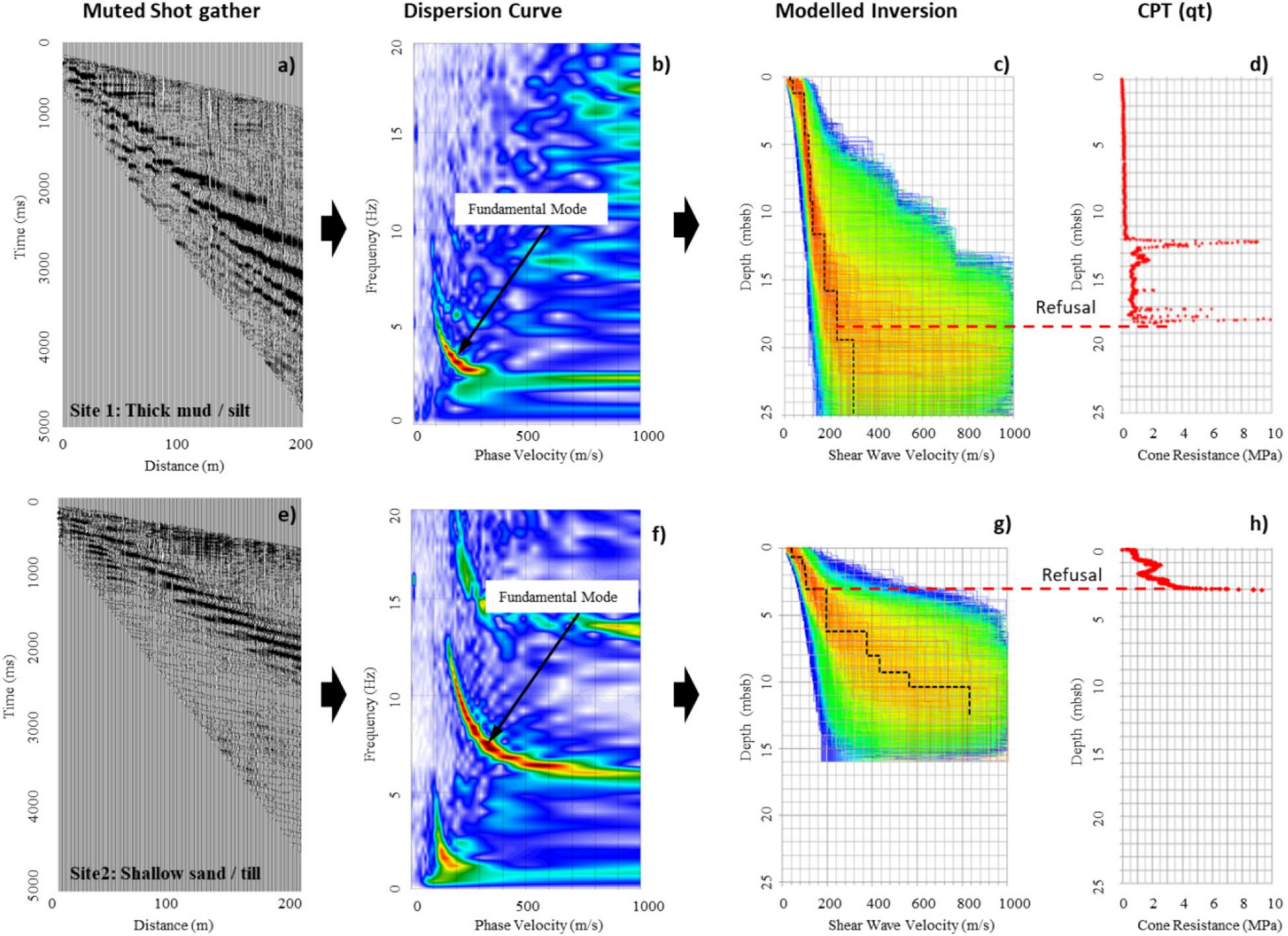
Processing flow for Site 1 (**a**–**d**) and Site 2(**e**–**h**), showing (a,e) muted shot gathers, (**b**, **f**) dispersion curve images, (**c**, **g**) inverted profiles, with best fit velocity models (dashed black lines), (**e**, **h**) correlation of modelled inversion to Cone Penetrometer Testing (Cone Resistance, qt).

The real time depiction of the DAS data, known as a Frequency Bandwidth Extracted (FBE) waterfall plot, was monitored to assess noise and data quality (Fig. 2). The FBE plot is generated by applying a Fast Fourier Transform to full bandwidth data and displaying the summed energy as a colour intensity plot. Figure 2a (frequency band 5 to 55 Hz) represents a 2-h 45 min portion of the recorded DAS data showing approximately the same duration of passive (upper) and active (lower) acquisition. The section of passive data highlighted in Fig. 2b shows 2 directions of ocean pressure waves travelling with apparent group velocities of c. 5.5 and 9 m/s. These pressure waves are also seen as steeply dipping, low velocity noise on the active shot records (Fig. 2c,d). Processing flows for data acquired at Sites 1 and 2 in the Dundalk Bay area are provided in Fig. 3. The multichannel shot gathers were muted (Fig. 3a,e) before being converted to a dispersion image using a 2D wave field-transformation method^27,28^. Dispersion curves were picked from the resulting frequency-phase velocity images (Fig. 3b,f) and extracted for inversion to produce a 1D V_s_-depth profile using a Monte-Carlo approach^29,30^ (Fig. 3c,g).

### Cable and source testing

**Figure 4:**
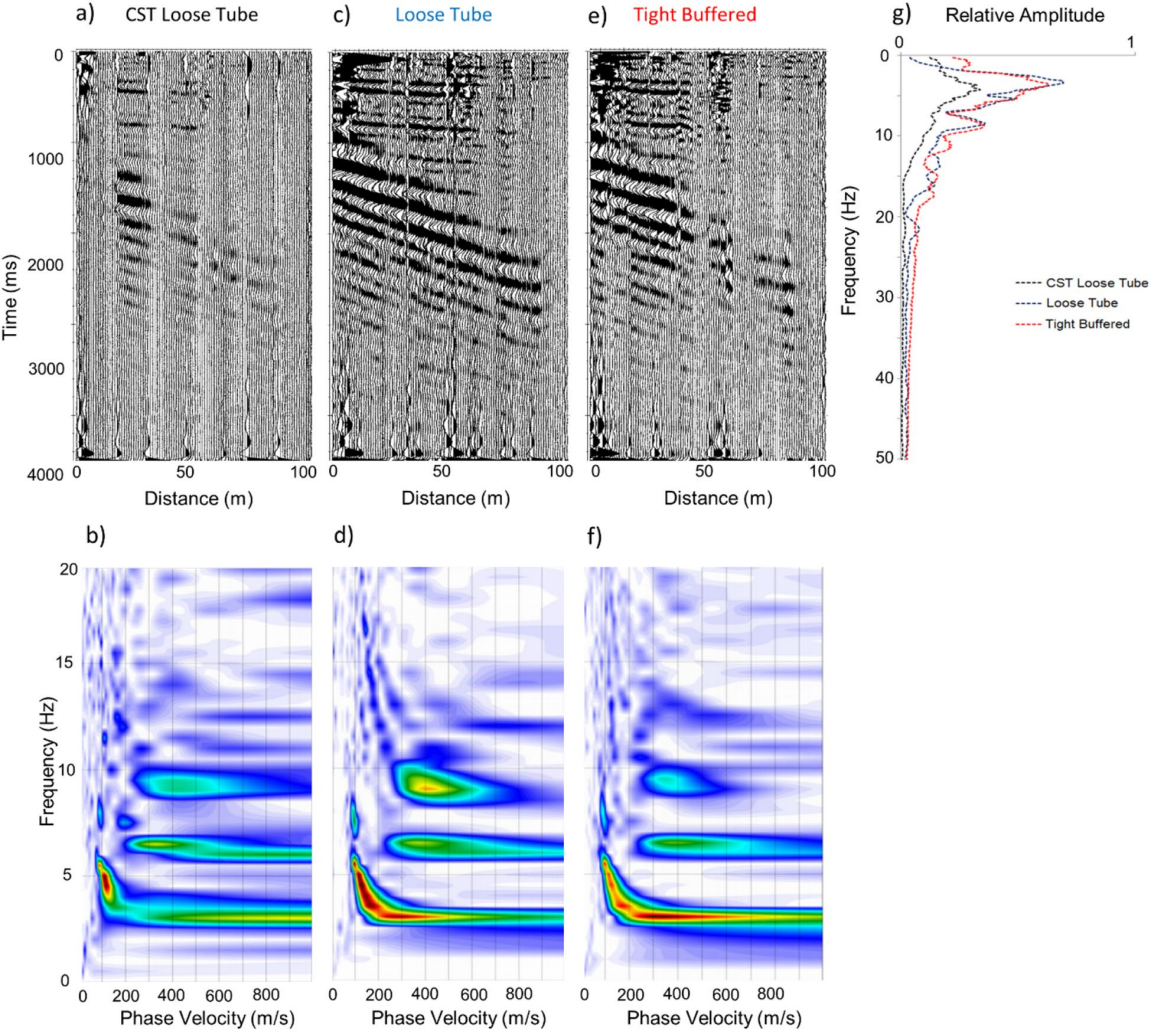
Seismic shot gathers and corresponding dispersion images acquired using (**a** & **b**) CST loose tube cable, (**c** & **d**) loose tube cable, (**e** & **f**) tight buffered cable. Relative amplitude vs frequency for each of the cable variants is shown in (**g**).

Due to the expected harsh environment associated with deployment and retrieval of cables in a marine environment, an armoured Corrugated Steel Tape (CST) loose tube cable was used for the majority of testing. In order to assess whether the reduced sensitivity of this cable had a meaningful affect on the dispersive properties of the Scholte wave, a comparison between different commercially available fibre cable variants was made. This involved the construction of a combined 300 m long hybrid cable consisting of the CST armoured loose tube, unarmoured loose tube and unarmoured tight buffered cable. The different fibre types were fastened together along their length with spliced returns at the distal end, resulted in a c. 1800 m continuous sensing element with 1 m channel spacing. The cables were carefully deployed on the seabed to avoid damaging the unarmoured variants. With this setup it was possible to carry out a direct comparison of the ground response from a single shot record (Fig. 4).

The shot records (Fig. 4a, c and d) show the armoured cable is the least sensitive, with a reduction in signal to noise ratio compared to the unarmoured variants, as would be expected. The tight buffered cable, which would be more sensitive to very low frequency strain, showed a slight reduction in quality of the shot response possibly as a result of the armouring properties of the tight buffered protection on the fibres. The phase velocity—frequency plots (Fig. 4b,d and f) show negligible difference between the unarmoured loose tube and tight buffered cables within the range of frequencies representing the fundamental mode Scholte wave response from the airgun source. The armoured CST loose tube cable showed a slight reduction in the higher end fundamental mode Scholte wave frequencies resolved (Fig. 4b), with an overall loss in amplitude over all frequency ranges (Fig. 4g).

**Figure 5:**
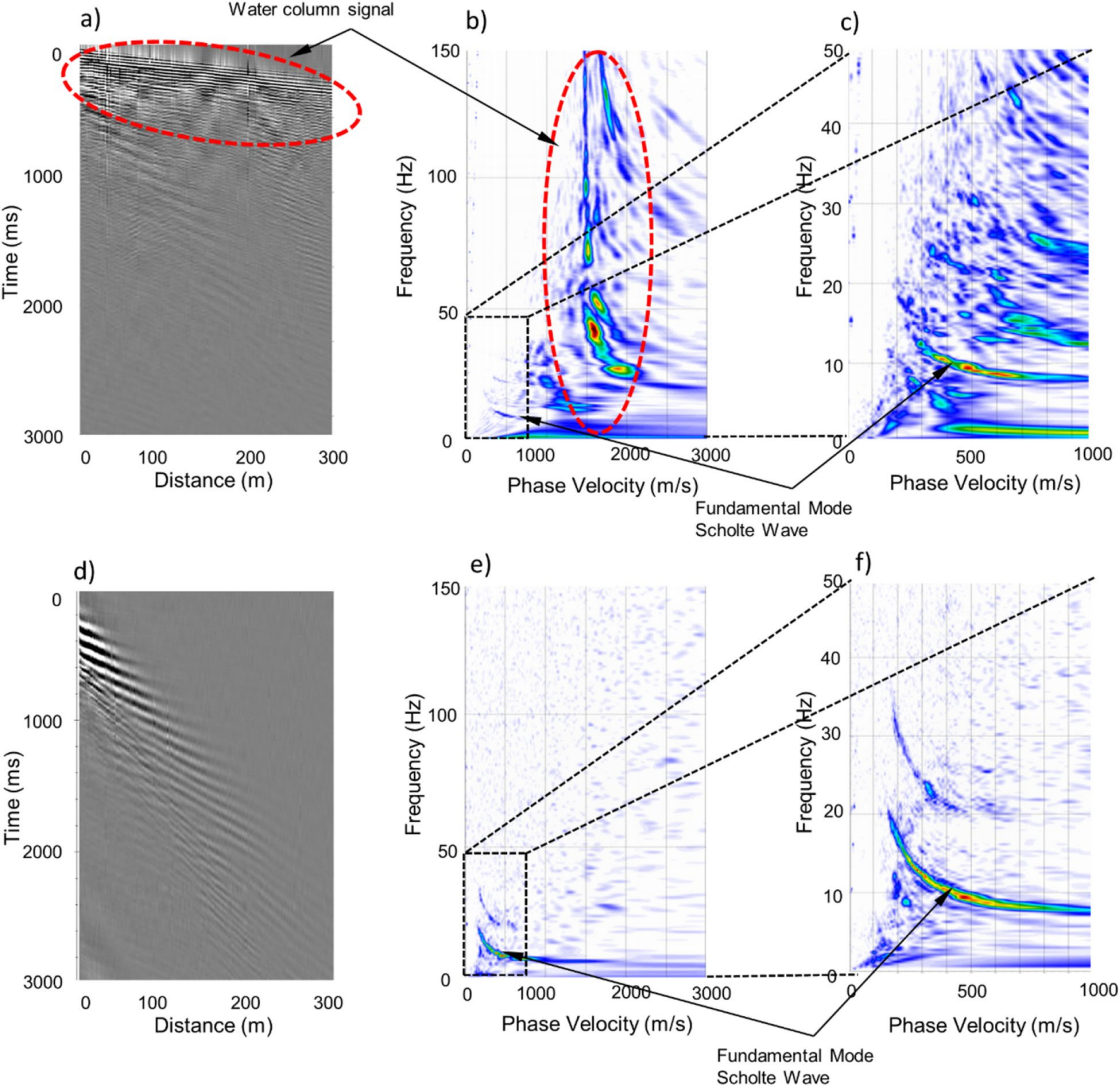
(**a**) Seismic shot gather and (**b**, **c**) corresponding dispersion images for airgun source. (**d**) Co-located seismic shot gather and (**e**, **f**) corresponding dispersion images for gravity corer source.

Due to the additional environmental & cost implications when using airgun sources, an assessment was also made as to the suitability of a gravity corer as an alternative source for near surface Scholte wave investigations. Airgun noise in the water column may cause harm to marine mammals^31,32^ and its use necessitates the presence of a MMO (Marine Mammal Observer) onboard the survey vessel, with survey operations paused when sea mammals are observed in the local area. The gravity corer, which is effectively a 600 kg marine weight drop that falls by gravity through the water column, is conventionally used for acquiring samples of near surface sediment. Figure 5 illustrates the considerably different seismic and dispersion image responses observed when using the airgun (Figs. 5a–c) compared to the gravity corer (Fig. 5d–f) at the same location. One of the main differences between the responses is the lack of significant energy transmitted through the water column from the gravity corer compared to an airgun source. The water column signal is also observed on the airgun dispersion image (Fig. 5b). It is apparent from the dispersion images that the fundamental mode Scholte wave can be resolved to a higher frequency when the gravity corer was used as the seismic source (Fig. 5f). In this case, the dispersion curve could be picked to a frequency of around 20 Hz, considerably higher than for the airgun image (Fig. 5c).

### Geophysical ground model

The correlation between inverted shear wave velocity and CPT data clearly shows the delineation between the depth of refusal and an increase in V_s_ to greater than c. 200 m/s (Fig. 3d,h). This is consistent with difficulties encountered with the use of CPT in medium dense to dense granular materials. The modelled inversions highlight the increased levels of uncertainty with depth associated with the analysis of the dispersion curve data, and the need to use all available geotechnical data to constrain the model, thereby reducing non-uniqueness and aiding interpretation.

**Figure 6:**
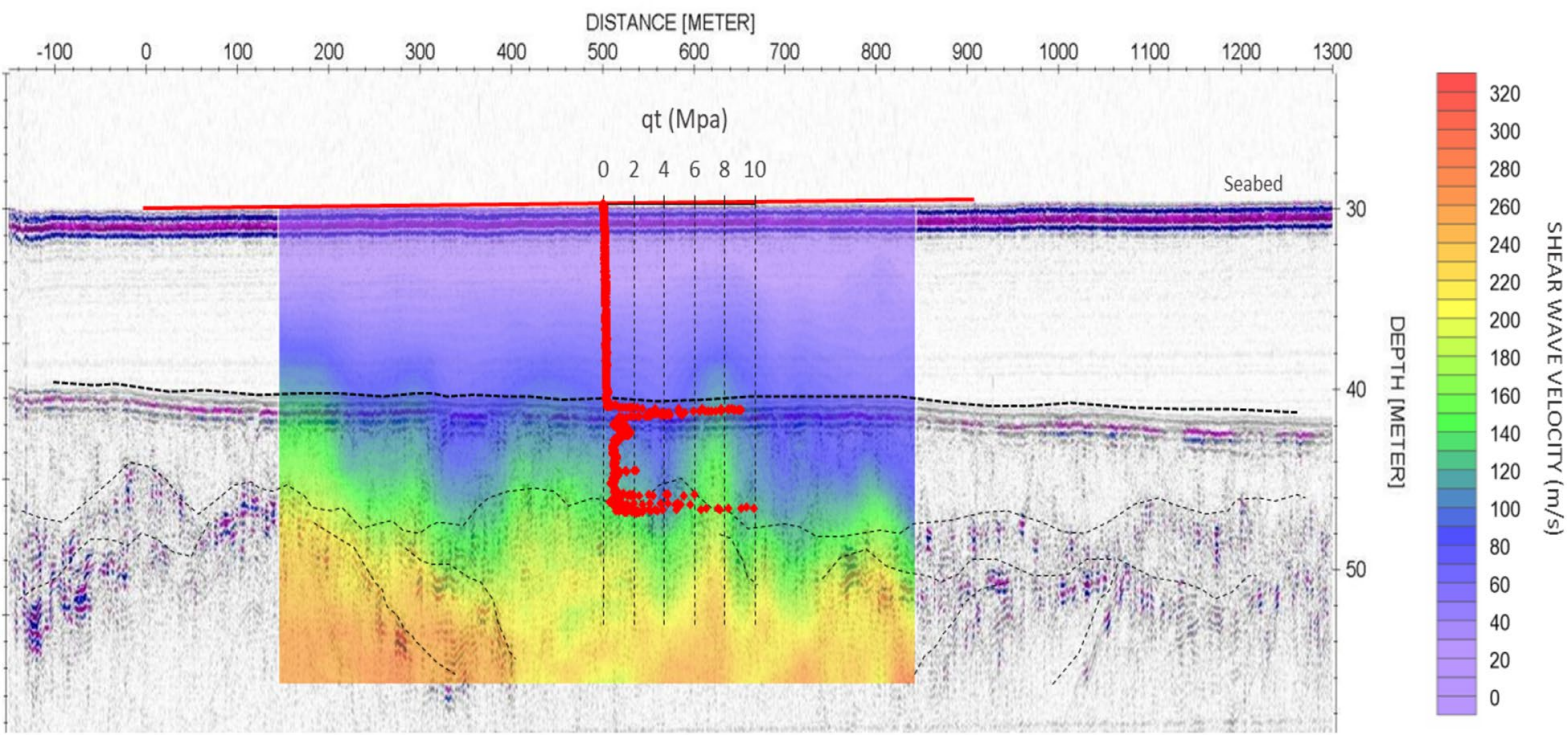
2D Shear Wave velocity profile (colour scale image) and CPT cone resistance, q_t_ (red line graph) overlaid on high resolution sparker reflection data (pseudo grey scale image) from Site 1.

An interpolated 2D V_s_ profile, combining individually inverted V_s_ profiles from Site 1, is provided in Fig. 6. This profile, along with a spatially referenced CPT profile, is overlain on sparker reflection data in this image. There is clear correlation between the location of sparker reflectors and corresponding spatial variations in V_s._ The upper c. 10 m sediment layer, with V_s_ of 80 to 150 m/s, and low cone tip resistance (q_t_), indicate very soft to soft muds/silts. These velocity ranges are consistent with the shallow returns from gravity cores which were logged as soft slightly sandy clayey SILT (Unit 1). Directly below these muds, joint interpretation of the CPT and V_s_ data suggests a lithological change to medium dense sands (Unit 2—150 to 200 m/s), with stiff, likely glacial till (Unit 3—greater than 200 m/s^33^), deposits forming the lowest resolvable layer.

Sub-bottom profiling is effective at determining acoustic layering but one of the limitations is the lack of velocity information relating to stiffness/density profiling. The lowest layer resolved in a sub-bottom profile section is often referred to as the acoustic basement and ideally would represent the top of bedrock but often this is not the case. By combining the sub bottom profile data with the DAS derived V_s_ profiles, it is possible to better understand the geological layering, while also enabling interpretation of the small strain shear stiffness, G_max_. In this case, DAS derived V_s_ profiling from Site 1, would indicate that the acoustic basement represents the transition from marine sediments to stiff glacial material rather than bedrock.

## Discussion

Distributed sensing of fibre optic cables has been shown to enable continuous, real-time measurements along the full length of a fibre optic cable. Using this approach, standard telecommunication optical fibres are interrogated by transmitting a temporally short light pulse and analysing the backscatter from microstructural variations naturally present within the fibre. In this study, we presented an approach for rapid acquisition of near-surface seismic data based on distributed acoustic sensing of fibre optic cables laid on the seabed. DAS has recently been successfully applied to passively monitor seismic events and the ambient noise wavefield from existing seafloor telecommunications cables for a number of applications^34–36^. Some studies have also indicated that passively generated Sholte waves from structures founded on the seabed may be of comparable frequencies to those measured actively in this study^37,38^. However, many of the areas where windfarms are planned, for example in the Irish Sea, are not located close to existing structures and the expected relatively low frequency nature of passively acquired Scholte wave data in these areas may not be suited for characterising the geophysical and geotechnical properties of the near surface, associated with the design of renewable energy infrastructure on the seabed. Conversely, the potential application of DAS, when combined with active seismic sources, is more suitable for high-resolution near surface offshore investigations^39,40^.

In terms of cable and source optimisation, despite the small reduction in measurable frequency range of the fundamental mode dispersion curve, the CST armoured cable appears to provide a reasonable compromise between performance and reliability in the harsh offshore environment. The benefits of marine weight drops, such as the gravity corer used in this survey, as Scholte wave seismic sources are also apparent due to their lack of impact on the water column. They are, therefore, more sympathetic in environmentally sensitive areas, such as Marine Special Areas of Conservation (SAC), where the use of airguns may have significant controls. It is also apparent that the gravity corer source has enabled the fundamental mode Scholte wave to be resolved to considerably higher frequencies than the airgun, thereby improving our ability to obtain near surface seismic properties of direct relevance to offshore foundation design. To operate the gravity corer, however, a boat is required with a winch powerful enough to lower and raise the corer, which may limit its use as a marine seismic source.

The DAS approach adopted in this study has shown great potential due to the high acquisition rates compared to other available technologies and its ability to accurately generate spatially extensive (km scale) 2D or 3D shear wave profiles. These data can also be carried out at sufficient resolution to support offshore infrastructure foundation design as well as potentially identifying suitable sub-sea cable routes. The application of this approach has the potential to considerably reduce design uncertainty and ultimately reduce levelised costs of offshore wind power generation. It is expected that further developments in the outlined methodology will secure DAS as an important investigative technique for the offshore geotechnical industry.

## Methods

The study relies on the measurement of acoustically induced strain determined from the interrogation of backscattered laser light from within a fibre optic cable to determine the shear wave profile of the shallow subsurface. The determination of shear wave profiles is characterised by a three phase process consisting of surface wave acquisition, processing and data inversion, and are discussed in turn below.

### Data acquisition

Two vessels were used during the data acquisition phase of the research. The RV Celtic Voyager was used as the acquisition platform and the Fionn Mac Cumhailll as the deployment / source vessel. An Optasense ODH4 Interrogator Unit (IU) was housed within the dry lab on board the acquisition vessel, along with a System Interface Unit (SIU) and a PC to integrate the GPS timing and trigger input and to capture the recorded data.

The fibre optic cable used was a Fibre Fox Uni-Tube Corrugated Steel Tape (CST) armoured loose tube variant with 4 individual single mode fibres. An OTDR was used prior to data collection to evaluate the fibre condition and check for any potentially damaging reflections at connections and spliced joints. Each pair of fibres had a spliced return at the distal end to give 2000 m of active sensing element. The cable was housed on a mechanical winch on the back deck of the Celtic Voyager. The Fionn MacCumhaill collected the distal end of the cable from the back deck and towed the cable along a predefined transect orientated to intersect the existing geotechnical data points. Once the full length of the cable was in position, the end was lowered along with a weight to fix to the seabed along with a second weight to mechanically isolate the retrieval buoy from the cable, preventing tugging noise from the buoy being transmitted along the fibre. The near end of the cable was then lowered with the same weight arrangement from the back deck of the Celtic Voyager. USBL transponders were also attached to the second weights to give real time positions of the cable ends and aid in the positioning of the gun boat in close proximity to the cable on the seabed.

The airgun was lowered to the seabed at each shot point location via a hoist mounted on the source vessel. This enabled the maximum energy to be generated and transferred to the sediment / water interface. The airgun used was a 12 cu in Sercel Mini G bolt gun operated at 2000 psi. The compressed air was delivered from standard 15 l dive cylinders, via a pressure manifold unit to regulate the pressure and also maintaining the pressure in the air lines during swapping of dive cylinders, thus optimising the amount of air being used. A GAPS Ultra Short Base Line (USBL) transponder was positioned 2 m above the airgun to give real time positioning of the gun string in relation to the cable position. The USBL system was monitored in real time on board the Celtic Voyager to ensure that the shots were located in close proximity to the cable position on the seabed. A Gisco Radio Trigger was used with the gun controller to send the T_0_ pulse to the SIU of the DAS recording system, on board the Celtic Voyager.

The DAS system interrogates the strain state of the fibre by transmitting temporally short light pulses in the order of 10 ns and assessing the backscatter from naturally occurring microstructural defects within the fibre. By careful time-gating of the back scattered light, the characteristics of the returning light, at specific locations, can be assessed. Using multiple successive pulses of light, the strain was interrogated several thousand times a second, allowing the detection of nanostrain induced by the interaction of the transmitted acoustic / elastic waves.

### Scholte wave data processing

Initially shot gather data were extracted from the DAS data using the T_0_ trigger files. End shot records with 100 and 200 traces and 5 s data length were selected to capture the Scholte wave offset data. This significantly reduced the file sizes improving data management and computation efficiency. A total of 35 shot gathers were extracted along the full length of the fibre sensing cable with an approximate shot spacing of 25 m. The data were temporally resampled to reduce the file size further and also filtered with a 2 Hz low cut filter to remove the low frequency strain component. Processing of the Scholte wave data was performed by selecting dispersion curves from a phase velocity-frequency spectra, generated using a wavefield transformation method^27,28,41–43^ in *Surfseis* (Kansas Geological Survey). For the purpose of this study the fundamental mode Scholte Wave dispersion data were used to generate the dispersion curves which were then converted into target files for input to the inversion stage of the process.

### Scholte wave inversion

The inversion process involves defining a velocity model(s) whose calculated dispersion curve best matches that from the measured Scholte Wave. To achieve this Monte Carlo forward modelling method was utilised using the Dinver module of the open-source software Geopsy^29,30^. The comparison fit between the measured and theoretical data was assessed by the use of a quantitative misfit value where the model with the lowest misfit was used as a new candidate model for the next iteration. 30 runs with 5,000 iterations were carried out, each using 50 random starting models. This resulted in the generation of 150,000 potential velocity models residing within the defined parameter space. The parameterisation was defined using 10 layers, each assigned a range of values for; thickness (H), shear wave velocity (V_s_), compression wave velocity (V_p_) and density (ρ). The average of the 50 best fit models were used for each of the 1D Vs profiles in order to generate the 2D Vs profile (35 shot locations). The position of the 1D profiles was based on the midpoint of the analysed shot gathers along the cable alignment. The 2D profile was generated using *Surfer* (Golden Software) with the krigging algorithm used for interpolation between 1D profiles.

### Sub bottom profiling

Sub-bottom profile data was gathered using a Geo-Source 400 sparker system on board the RV Celtic Voyager as part of the Irish Sea Marine Assessment (ISMA) survey^44^.

The sparker system consisted of a Geo-Spark 1500 kJ power supply operating predominantly at a frequency of between 0.5 and 2 kHz. The unfiltered return signal was recorded using a Geo-Sense single channel hydrophone array. Both the sparker source and hydrophone were towed 25 m behind the vessel, off opposite corners of the back deck with a lateral separation of c. 8 m. Data were collected at a towing speed of c. 4 knots and recorded using the Coda software on-board the Celtic Voyager. The single channel data were recorded in proprietary Coda and SEGY formats with integrated navigation. The raw data were imported into the ReflexW software prior to processing. A seabed static correction was applied based on the available bathymetry data. A 2-trace running average and 4-trace stack was applied to supress the random background noise. Horizons were picked manually, and seismic depths were converted from two-way travel time to metres using an acoustic internal velocity of 1600 m/s, expected to correspond to the interval velocity of the upper soft sediments.

### Cone penetrometer testing (CPT)

The CPT is a routinely used in situ method for determining the geotechnical engineering properties of soils and delineating soil stratigraphy^45–47^. The CPT data presented in this study were collected as part of the CE14001 survey on board the RV Celtic Explorer using the Geotechnical Offshore Seabed Tool (GOST)^48^. The GOST system is a seabed mounted ‘push tool’ equipped with a hardened stainless steel instrumented cone tip which is pushed into the sediments at a controlled rate. The instrumentation records tip resistance, sleeve friction, differential pore pressure, and inclination, acceleration and heat conductivity with a resolution of 0.06 MPa allowing for a range up to 120 MPa. The cone tip has a cross-sectional area of 5 cm^2^. The GOST system has an operational weight of between 2 and 8 tonnes depending on the addition of weighted plates for extra stability. It has 8 tonnes of hydraulic push power with variable hydraulic pressure of 0–20 MPa. Exact control on push velocity during penetration allows for data to comply to the highest international standards including DIN 4904 and ISO 22476-1:2012 requirements. The interface was a digital one of industrial RS485 BUS using direct A/D converting of a measured variable with overvoltage and reverse protection. Raw CPT data was calibrated using standard methods according to^45^. In addition to the primary CPT parameters of cone resistance, sleeve friction and pore pressure, secondary parameters including undrained shear strength (c_u_) can be calculated using empirical approaches^45,49,50^.

## Data Availability

The datasets used and/or analysed during the current study available from the corresponding author on reasonable request.
